# Quadrilateral plate classification program of acetabular fractures based on three-column classification: a three-dimensional fracture mapping study

**DOI:** 10.1186/s13018-024-04783-z

**Published:** 2024-05-16

**Authors:** Ruihan Wang, Songtao Jiang, Wei Wang, Yingqiu Yang, Lei Zhang, Guoyou Wang

**Affiliations:** 1 Department of Rehabilitation, Yibin Integrated Traditional Chinese and Western Medicine Hospital, Yibin, China; 2 School of Physical Education, Southwest Medical University, Luzhou, China; 3 School of Clinical Medicine, Southwest Medical University, Luzhou, China; 4 Department of Orthopedics, The Affiliated Traditional Chinese Medicine Hospital, Southwest Medical University, 182 Chun Hui Road, Luzhou, 646000 Sichuan province China; 5 Center for Orthopedic Diseases Research, The Affiliated Traditional Chinese Medicine Hospital, Southwest Medical University, Luzhou, China; 6 Luzhou Key Laboratory of Orthopedic Disorders, Southwest Medical University, Luzhou, 646000 China

**Keywords:** Acetabular fracture, Three-column classification, Quadrilateral plate, Fracture mapping, Fracture lines, Heat map

## Abstract

**Background:**

A new classification system for acetabular fractures has been proposed in recent years, which is called the 3-column classification. However, this system does not provide information regarding quadrilateral plate fractures. To address this issue, we utilized three-dimensional (3D) fracture line mapping and heat map to analyze the link between the 3-column classification and quadrilateral plate fractures.

**Methods:**

We collected CT scan data from 177 patients who had been diagnosed with acetabular fractures. Additionally, we utilized a CT scan of a healthy adult to generate a standard acetabular model. We utilized the collected CT data of the fracture to create a 3D model and subsequently reduced it. We then matched each acetabular fracture model with the standard acetabular model and mapped all of the fracture lines to the standard model. 3D fracture lines and heat maps were created by overlapping all fracture lines. Fracture characteristics were then summarized using these maps.

**Results:**

This study analyzed a total of 221 acetabular fractures. The most frequently observed fracture type, based on the three-column classification, was A1.2, which corresponds to fractures of the anterior column. In contrast, the least common type of fracture was A4, which represents fractures of the central wall. It was noted that quadrilateral plate fractures were frequently observed in fractures classified as type B and C according to the three-column classification.

**Conclusions:**

Among the three-column classification, the QLP fractures are commonly observed in type B and C. It is important to carefully identify these fractures during the diagnostic process. Therefore, based on the three-column classification, we have amalgamated quadrilateral plate fractures and formulated a classification program for acetabular fractures.

## Introduction

Due to the aging population, the occurrence of hip fractures has risen quickly. From 1980 until 2007, the occurrence of hip fractures in older individuals increased by 2.4 times. Within these hip anterior fractures, quadrilateral plate (QLP) fractures account for a high percentage at 50.8% [1]. These fractures are known for their high incidence and disability and cause a significant burden on patients and society [2].

The QLP, a crucial component of the acetabulum and located at the bottom of its bowl, is conventionally seen as the medial surface of the acetabulum and bears the majority of the articular surface [3]. However, the traditional Judet-Letournel classification system does not clearly define the QLP fracture. The Cairo University Hospitals (CUH) classification system is a frequently used method for QLP fracture classification [4]. However, it has not been integrated with the “column” fracture theory, which has resulted in some limitations. In addition, other defects have been proven in the Judet-Letournel classification system [5, 6]. It defines fractures of the anterior columns as discontinuities of the iliopectineal line based on the 2-column concept and defines fractures of the posterior columns as discontinuities of the ilioischial line [7, 8]. However, fractures that affect both iliopectineal and ilioischial lines, such as transverse, T-type, and transverse plus posterior wall fractures, do not fall under the classification of both-column fractures [6, 8–10]. Based on the shortcomings of the above Judet-Letournel classification system, Zhang et al. [11] proposed the “3-column” theory for acetabular fractures in 2019. According to the growth period of the hemipelvis, the three-column classification of the acetabulum involves the ilium branch forming the roof, the pubis branch forming the front column, and the ischium branch forming the back column [11]. The 3-column classification provides a clearer and more inclusive way of categorizing acetabular fractures, solving the issue of conceptual confusion. It also has high credibility and repeatability, making it easier for orthopedic surgeons to understand and apply to diagnosis and treatment. However, whether the three-column classification can be combined with the CUH classification to further increase the application of this classification remains unknown.

The virtual computed tomography (CT) modeling software generates three-dimensional (3D) fracture models, which facilitates the analysis of the fracture appearance in a more comprehensive way. By integrating 3D fracture line mapping with a heat map based on the CT data, more information can be obtained regarding fracture patterns and morphology [12]. Therefore, we plan to observe the acetabular and QLP fractures using 3D fracture line mapping and heat maps with the 3-column classification.

This study aims to examine the link between the 3-column classification system and the QLP fracture by analyzing 3D fracture line mapping and heat map. This study hopes to advance the diagnosis and treatment of patients with acetabular fractures by optimizing the classification system.

## Materials and methods

### Specimen selection

Computed tomography (CT) data for 177 patients diagnosed with acetabular fracture at the Affiliated Hospital of Traditional Chinese Medicine, Southwest Medical University, between 2018 and 2022, were retrospectively analyzed. The inclusion criteria were (1) age ≥ 18 years old; (2) fractures and any noticeable skeletal deformities are observed easily. The exclusion criteria were (1) a prior history of acetabulum surgery, (2) pathological or chronic fracture, and (3) a history of ipsilateral acetabulum deformity. Due to the majority of collected cases being male and with an average age and standard deviation of 52.04 ± 17.19 years old, we decided to use a healthy adult male model who was 54 years old as the standard model (Table 1). We obtained CT data from this healthy adult male and used it to develop a standard acetabular model.

**Table 1:** General information of patients

Character	Patients (*n* = 177)
Male	101 (57.1%)
Female	76 (42.9%)
18–30 years old	24 (13.6%)
31–50 years old	54 (30.5%)
51–70 years old	79 (44.6%)
71 years old - above	20 (11.3%)
Average age	52.04 ± 17.19
Lift side injury	69
Right side injury	64
Bilateral injury	44
Number of acetabular fractures	221

### 3D fracture mapping and heat map

Fracture CT scans were taken using a 128-slice spiral computed tomography scanner (Siemens Somatom, Germany), and the data was exported in a DICOM format. 3D reconstruction was done using Mimics (21.0, Materialise, Belgium). Each patient’s data was saved as a new project file using the “New project wizard” tool, and the DICOM format file was imported. A grayscale range of 226–3071 Hounsfield units (HU) was used as the threshold range for the whole bone, but for some bones with inapparent grayscale values (osteoporosis, etc.), we adjusted the HU range to determine the optimal threshold. Despite advancements in segmentation techniques, manual segmentation was necessary for some irregular fracture regions. In the case of intricate models, two individuals (RW and SJ) manually segmented, modeled, and validated the corresponding models independently. For displaced fractures, we used the N-Point Alignment function of the reverse engineering software (Geomagic Wrap 2021, 3D Systems, United States), and if there were cases of poor displacement, we used the Using Best Fit Alignment function to re-displace the fracture. All the models that have been created and saved in STL format. Additionally, the right acetabulum was used as the standard model in this case, so all the models of the left acetabulum were mirrored to align with the corresponding structures present on the right side.

The 3D models of the acetabular fractures that were created were imported into 3-matic (13.0, Materialise, Belgium). We utilized the “N points registration” to match and line up the fracture model with the standard model. After that, the fracture line was traced and marked onto the standard model. All fracture lines that were found in acetabular fractures were indicated on a 3D standard model of the acetabulum to create fracture line mapping graphs. The fracture lines mapping graphs were saved in TXT format, indicating coordinates with a high accuracy of 0.0001 mm, presented in (x, y, z) form.

The fracture lines mapping graphs were imported into the E-3D Digital Medical Platform (Central South University, Changsha, China). A heat map is to be generated by converting fracture lines based on their frequency of occurrence in every position of the 3D model. Distance weighting will be utilized during the calculations to factor in possible human error and differences in the acetabular morphology, with a radiation distance of 5 mm per line.

### Data analysis

This study presented patient demographics and fracture characteristics using mean values and standard deviations (M ± SD) for continuous variables and counts and percentages (%) for categorical variables. The distribution, intensity, and frequency of fracture lines seen through the fracture mapping model were described using qualitative analysis.

## Results

The study involved 177 patients, with 57.1% being male and 42.9% being female and an average age of 80 years. Patients aged 18–30 accounted for 13.6%, while those aged 31–50, 51–70, and 71-above represented 30.5%, 44.6%, and 11.3%, respectively. Out of the total patients, 69 had injuries on their left side, 64 on their right side, and 44 suffered from injuries on both sides (Table 1).

### Three-column classification for acetabular fractures

**Fig. 1:**
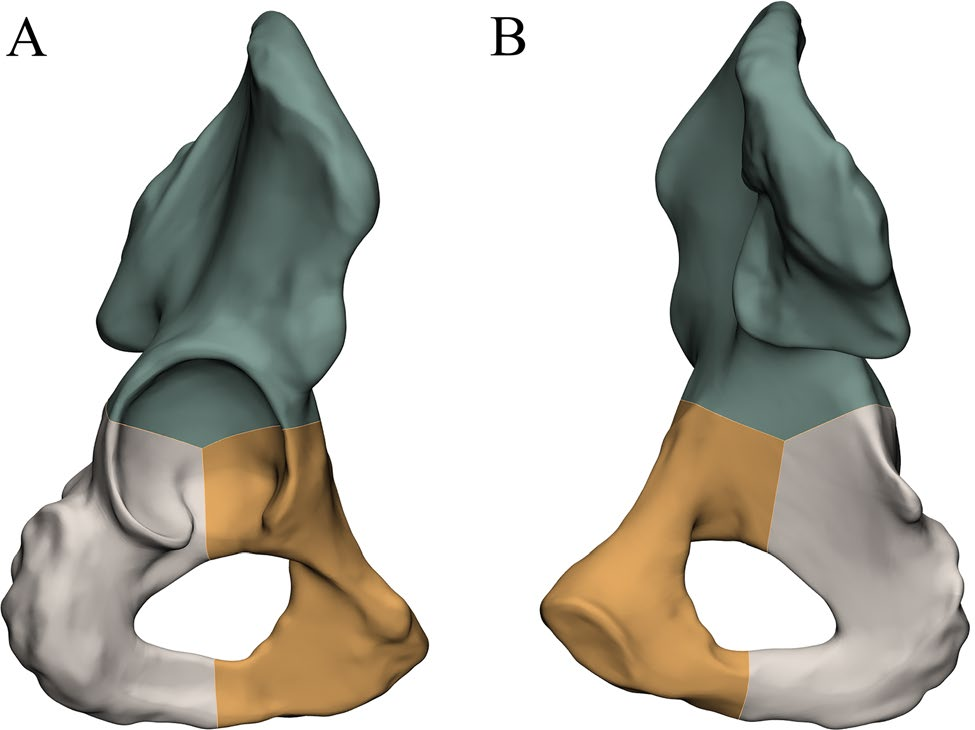
The components of the acetabulum are indicated with varying colors according to the 3-column classification: anterior column (orange), posterior column (gray), and roof column (green). **A**: anterior view; **B**: posterior view

According to the definition of the 3-column classification system by Zhang et al. [11], the anterior column, posterior column, and roof column are composed of the pubis, ischium, and ilium, respectively. The areas between the roof and anterior columns, roof and posterior columns, and anterior and posterior columns are known as the anterior wall, posterior wall, and medial wall, respectively. Lastly, the roof wall refers to the weight-bearing section of the acetabulum formed by the ilium (Fig. 1).

According to the classification system, Type-A fractures involve a single wall or column of the acetabulum. A1.1 (12; 5.4%), A1.2 (54; 24.4%), and A1.3 (12; 5.4%) refer to different types of anterior wall or column fractures. A2.1 (20; 9.0%), A2.2 (9; 4.1%), and A2.3 (5; 2.3%) refer to different types of posterior wall or column fractures. A3.1 (2; 0.9%), A3.2 (15; 6.8%), and A3.3 (5; 2.3%) represent different types of roof wall or column fractures, while A4 (3; 1.4%) refers to an isolated medial wall of the acetabulum (Table 2).

**Table 2:** Three-column classification for acetabular fractures

Classification			No. of fractures (*n* = 221)
A (single column or wall detachment)	A1 (anterior column or wall)	A1.1 (anterior wall)	12 (5.4%)
		A1.2 (anterior column)	54 (24.4%)
		A1.3 (complicated anterior column)	12 (5.4%)
	A2 (posterior column or wall)	A2.1 (posterior wall)	20 (9.0%)
		A2.2 (posterior column)	9 (4.1%)
		A2.3 (complicated posterior column)	5 (2.3%)
	A3 (roof column or wall)	A3.1 (roof wall)	2 (0.9%)
		A3.2 (roof column)	15 (6.8%)
		A3.3 (complicated roof column)	5 (2.3%)
	A4 (medial wall)	-	3 (1.4%)
B (2-column detachment)	B1 (roof-anterior columns)	B1.1 (intact roof-anterior columns)	6 (2.7%)
		B1.2 (separated roof-anterior columns)	5 (2.3%)
		B1.3 (complicated roof-anterior columns)	4 (1.8%)
	B2 (anterior-posterior columns)	B2.1 (intact anterior-posterior columns)	27 (12.2%)
		B2.2 (separated anterior-posterior columns)	16 (7.2%)
		B2.3 (complicated anterior-posterior columns)	7 (3.2%)
C (3-column detachment)	C1 (elementary 3-column)	-	5 (2.3%)
	C2 (3-column with posterior wall)	-	5 (2.3%)
	C3 (complicated 3-column)	-	9 (4.1%)

Type-B fractures involve two columns or walls. B1.1 (6; 2.7%), B1.2 (5; 2.3%), and B1.3 (4; 1.8%) refer to different types of roof-anterior column fractures. B2.1 (27; 12.2%), B2.2 (16; 7.2%), and B2.3 (7; 3.2%) refer to different types of anterior-posterior column fractures (Table 2).

Type-C injuries involve three columns or walls of the acetabulum. C1 (5; 2.3%) represents a simple 3-column fracture, C2 (5; 2.3%) represents a 3-column fracture with posterior wall detachment, and C3 (9; 4.1%) represents a complicated 3-column fracture (Table 2).

### 3D fracture line mapping and heat map

**Fig. 2:**
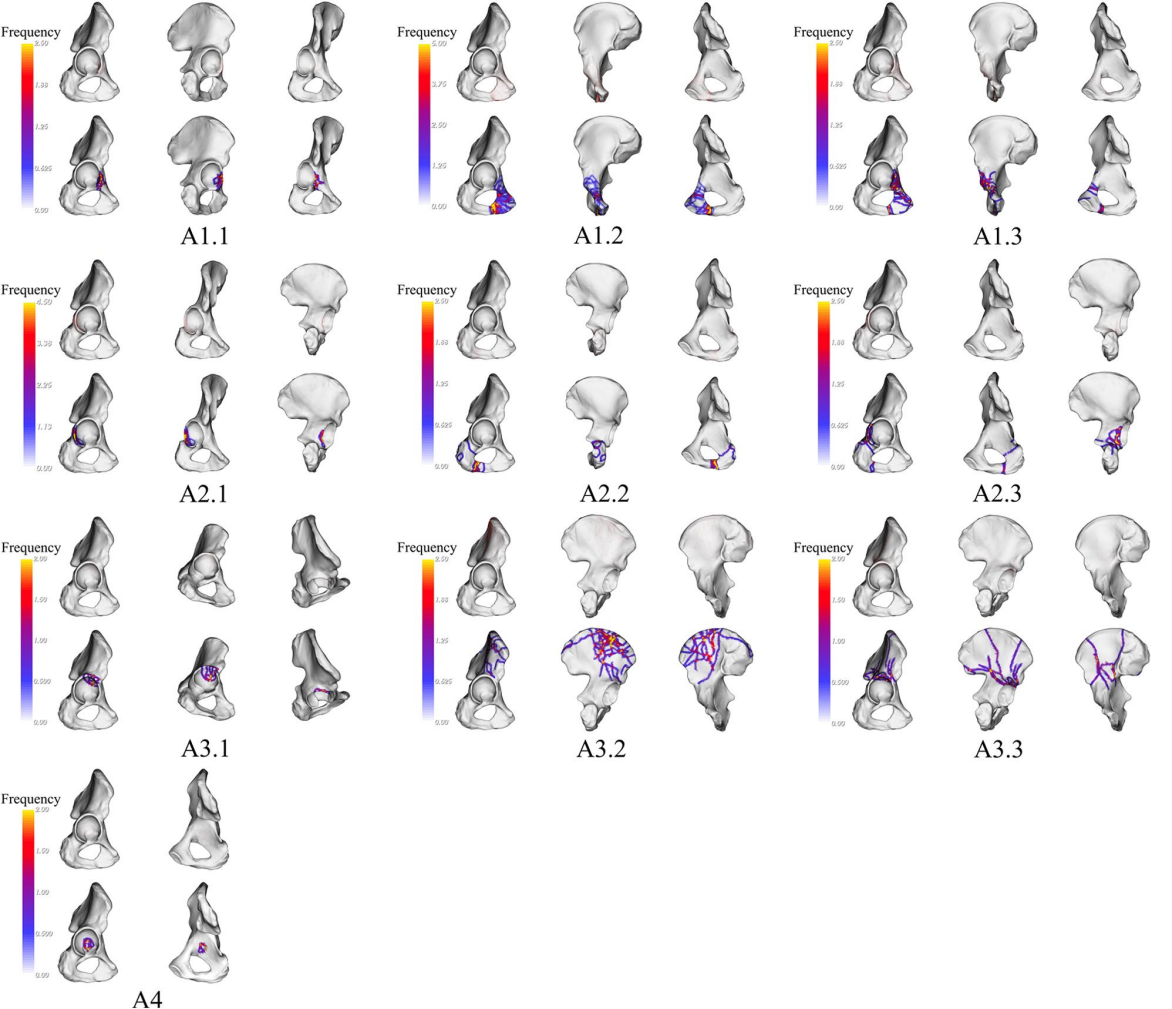
Visualization of fracture lines and heat maps for Type-A fracture. Each red line represents the position of each fracture. The heat map displays a transition of colors from yellow to gray, illustrating the fluctuation in the occurrences of fracture lines. As the color shifts from yellow to gray, it represents a decline in the frequency of fracture lines, moving from a higher occurrence to a lower occurrence

Fracture lines and areas of increased hotspots associated with A1.1 are primarily located on the lower end of the anterior wall. A1.2 shows fracture lines and hotspots concentrated at the junction of the lower end of the acetabulum anterior column and the posterior column. A1.3 exhibits fracture lines and hotspots mainly at the junction of the anterior column and the anterior wall, as well as the junction of the lower end of the acetabulum anterior column and the posterior column. A2.1 shows fracture lines and hotspots primarily concentrated on the lower end of the posterior wall. A2.2 exhibits fracture lines and hotspots in the lower part of the posterior column and at the junction of the lower end of the acetabulum anterior column and the posterior column. Fracture lines and hotspots of A2.3 are mainly concentrated at the junction of the posterior wall and the posterior column. A3.1 shows fracture lines and hotspots mainly on the outer side of the anterior wall. A3.2 exhibits fracture lines and hotspots mainly in the upper part of the roof column. A3.3 exhibits fracture lines and hotspots mainly at the junction of the roof wall and the roof column. A4, on the other hand, displays fracture lines and hotspots primarily concentrated in the middle part of the medial wall (Fig. 2).

**Fig. 3:**
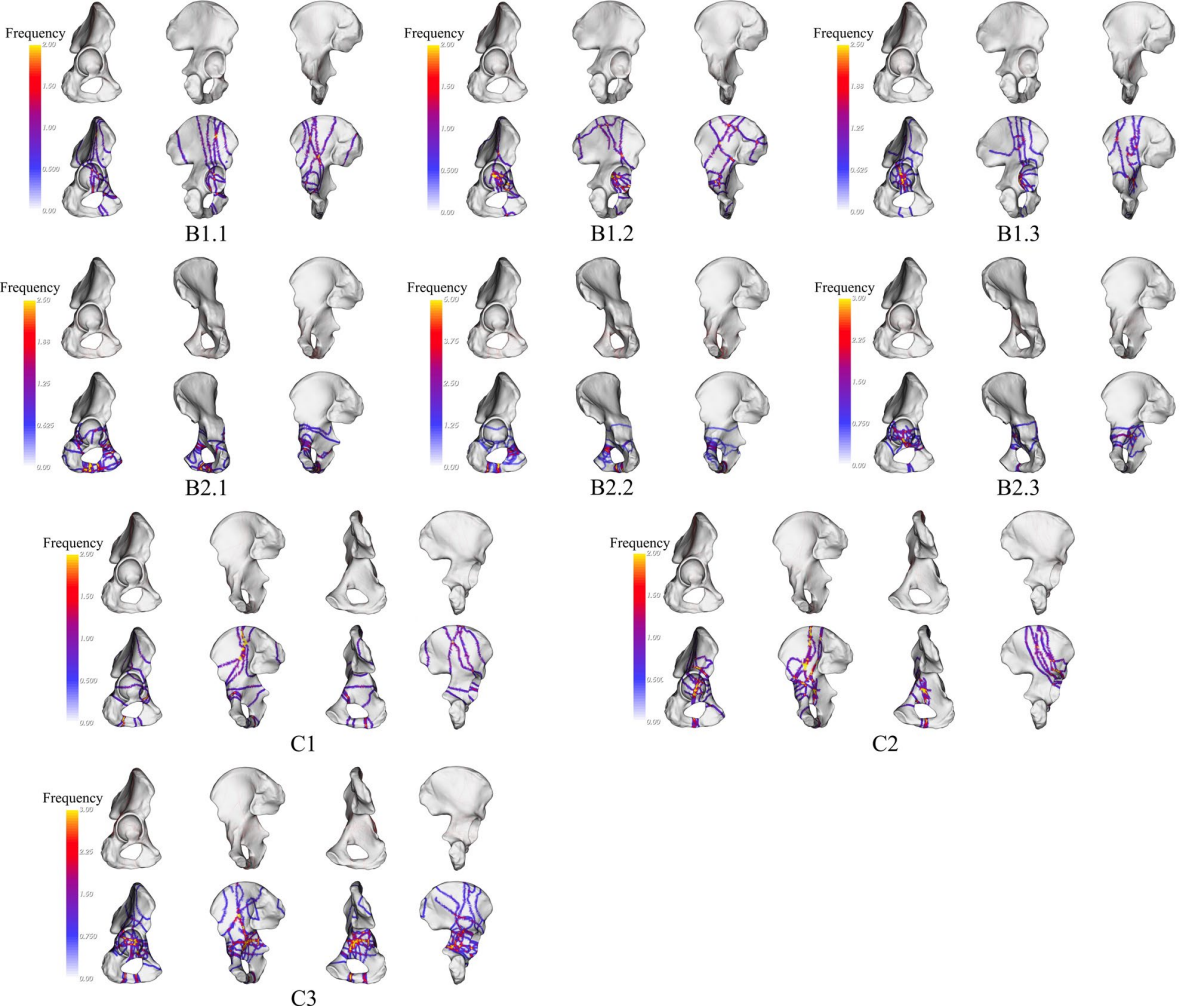
Visualization of fracture lines and heat maps for Type-B and Type-C fracture. Each red line represents the position of each fracture. The heat map displays a transition of colors from yellow to gray, illustrating the fluctuation in the occurrences of fracture lines. As the color shifts from yellow to gray, it represents a decline in the frequency of fracture lines, moving from a higher occurrence to a lower occurrence

The fracture lines and heat maps of B1.1 are primarily focused on the junction between the medial and anterior walls. B1.2 exhibits fracture lines and heat maps concentrated in the medial wall, and the lower portion of the anterior wall. B1.3 shows a concentration of fracture lines and heat maps in the middle part of the medial wall. B2.1 displays fracture lines and heat maps concentrated at the junction of the anterior wall and anterior column, as well as at the junction of the lower end of the acetabulum anterior column and the posterior column. B2.2 exhibits a concentration at the junction of the anterior column and anterior wall, as well as at the junction of the lower end of the acetabulum anterior column and the posterior column. B2.3 shows a concentration primarily at the junction of the medial and anterior wall (Fig. 3).

C1 displays a concentration of fracture lines and heat maps at the junction of the anterior wall and anterior column, as well as at the junction of the lower end of the acetabulum anterior column and the posterior column. C2 exhibits a concentration in the medial, the roof column, and at the junction of the roof wall and roof column. C3 shows a concentration mainly in the middle part of the medial wall (Fig. 3).

### Correlation of quadrilateral plate fractures in the three-column classification

**Fig. 4:**
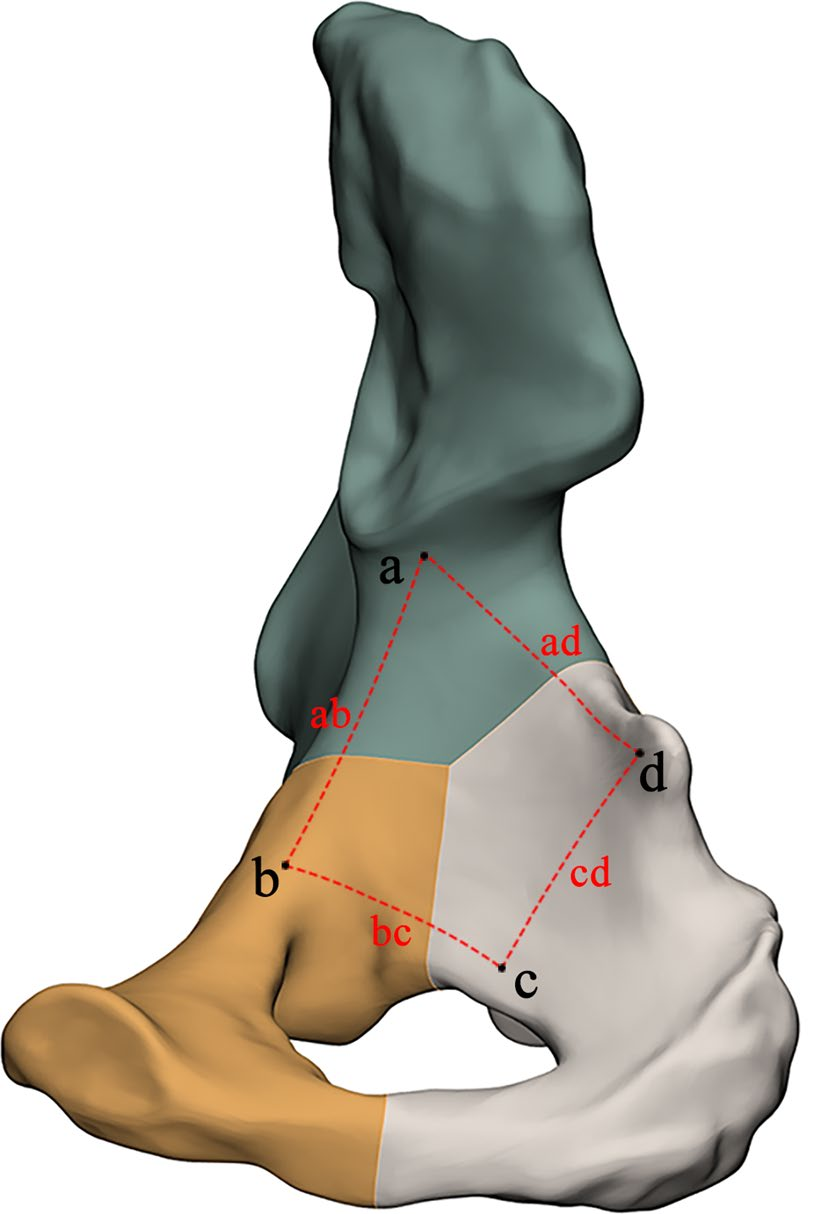
QLP four-point classification

Based on the 3-column classification system, we further analyzed QLP fracture. According to the definition of the four-cornered area by Elnahal et al. [4], Yang et al. [13] utilized four points, namely “a, b, c, d”, to denote the lower edge of ilium, obturator groove, rear edge of the obturator ring, and ischial spine, respectively. The region formed by the four lines (ab, bc, cd, ad) represents a quadrilateral region (Fig. 4). We refer to this classification of quadrilaterals known as the QLP four-point classification.

**Fig. 5:**
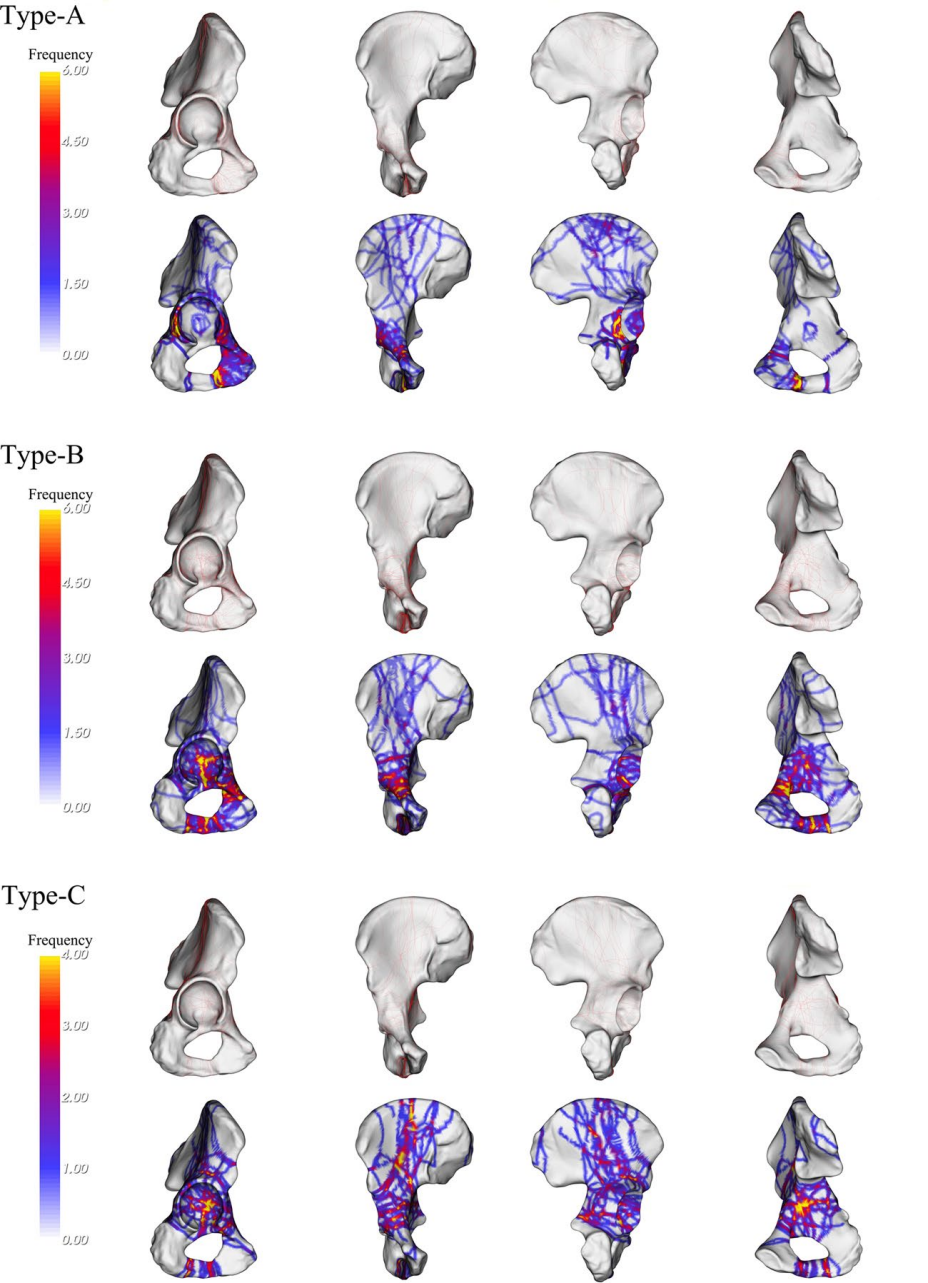
Visualization of fracture lines and heat maps for three total fracture types. Each red line represents the position of each fracture. The heat map displays a transition of colors from yellow to gray, illustrating the fluctuation in the occurrences of fracture lines. As the color shifts from yellow to gray, it represents a decline in the frequency of fracture lines, moving from a higher occurrence to a lower occurrence

According to this classification, QLP fractures have been classified and summarized based on the three-column classification system (Table 3). When looking at the fracture from an anterior view, Type-A fractures are mainly found at posterior wall and the junction of the lower end of the acetabulum anterior column and the posterior column. On the other hand, Type-B and Type-C fractures are predominantly located at the medial wall and the junction of the anterior wall and anterior column. When viewing the fracture from a posterior perspective, Type-A fractures are uncommon in QLP fractures, whereas Type-B and Type-C fractures are more common (Fig. 5).

**Table 3:** Quadrilateral plate four-point classification in the three-column classification

Classification	A4	B1.1	B1.2	B1.3	B2.1	B2.2	B2.3	C1	C2	C3	All
ab	-	-	-	3	-	-	3	-	1	2	9
bc	2	-	-	2	-	2	1	-	-	-	7
cd	-	-	-	-	-	-	-	-	-	-	-
ad	-	-	-	-	-	-	1	-	-	1	2
ab + bc	-	4	3	4	-	1	2	3	6	4	27
ab + cd	-	-	-	-	-	-	-	-	-	-	-
ab + ad	-	-	-	-	2	1	2	2	-	4	11
bc + cd	-	-	-	-	-	-	1	1	-	-	2
bc + ad	-	-	-	-	-	-	-	-	-	-	-
cd + ad	-	-	-	-	-	-	-	-	-	2	2
bc + cd + ad	-	-	-	-	-	-	-	-	-	1	1
ab + cd + ad	-	-	-	-	-	-	-	-	-	-	-
ab + bc + ad	-	-	-	-	-	-	-	-	-	-	-
ab + bc + cd	-	-	-	-	-	-	-	-	-	1	1
all	2	4	3	9	2	4	1	6	7	15	62

## Discussion

In this study, we utilized 3D fracture line mapping and thermography techniques to examine and describe the fracture line patterns of acetabular fractures. We started by presenting a summary and visualization of the 3-column classification system for these fractures. Building on this classification, we observed and documented the incidence of QLP fractures in three total fracture types. By analyzing QLP fractures within the 3-column classification, we observed that they are frequently seen in Type-B and Type-C fractures.

Surgical fixation is typically the primary approach used to treat most acetabular fractures. The objective is to maintain stability and alignment of the pelvis, achieve optimal functional outcomes, and minimize the risk of developing osteoarthritis [14]. However, there is currently no surgical approach that can be applied to all types of acetabular fractures. Therefore, knowing the type of acetabular fracture is crucial for achieving anatomical reduction of the joint. The 3-column classification system for acetabular fractures is based on the division of the acetabulum into “three columns” and “four walls” [11]. This system has been found to possess a higher level of consistency between observers and within the same observer compared to the Judet-Letournel classification. Furthermore, it encompasses a larger variety of fracture types. In our study, we have discovered that the 3-column classification system can be applied universally to all cases we collected. It can be used as a supplementary tool alongside the traditional Judet-Letournel classification.

The QLP is a 3D structure that extends from the inner edge of the acetabulum to the medial side. It is located near important structures such as the femoral artery, femoral vein, femoral nerve, spermatic cord/round ligament of the uterus, pudendal neurovascular bundle, and bladder. Improper operation can cause damage to these vital tissues [15]. Although the classification of acetabular fractures does not fully include QLP fractures, from the aspect of the 3-column classification, some of them belong to the anterior column, some belong to the posterior column, and some belong to the roof column. The roof of the acetabulum is responsible for supporting most of the body’s weight, and the QLP is a crucial part of this area that helps keep the femoral head in contact with the main weight-bearing area. This prevents the femoral head from moving inward and sticking out into the pelvic cavity [16]. However, if the QLP fractures, it can disrupt the proper relationship of the joint and lead to an imbalance in mechanical forces between the acetabulum and the femoral head. This imbalance can lead to reduced weight-bearing surface on the joint and result in stress concentration [17].

**Fig. 6:**
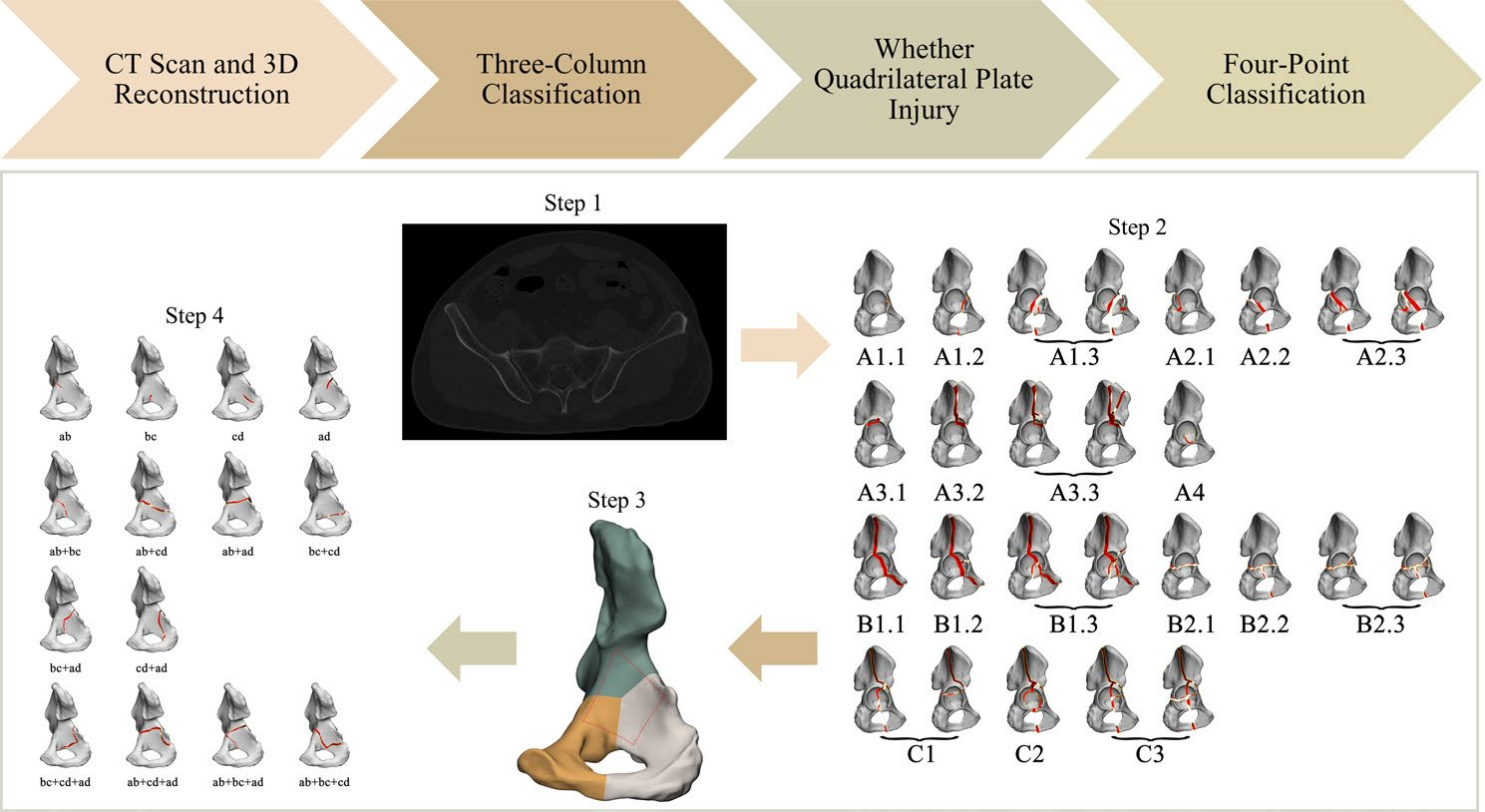
Classification program for acetabular fractures

In our study, the common QLP fractures observed were ab, bc, ab + bc, and ab + ad, involving the anterior wall, medial wall, anterior column, and posterior wall. Most cases of QLP injuries showed varying degrees of medial displacement of the femoral head. In addition, some cases of incomplete QLP fractures affecting the anterior and posterior columns were also identified. In 2023, Chen et al. [18] evaluated QLP fractures using a new classification system. Their classification was based on the anterior or posterior column fractures of the Judet-Letournel classification and the partial or complete separation of QLP from the columns. However, the Judet-Letournel classification system still has limitations in classifying acetabular fractures [11], and currently, QLP is often adopted as a separate classification system [4]. Considering the limitations of the Judet-Letournel classification, we propose a 3-column classification as the basis for evaluating QLP fractures. Our study, however, has revealed that not all fractures near the acetabular fossa result in the involvement of the QLP. Additionally, the inclusion of QLP fracture classification alongside the existing three-column classification would lead to confusion and hinder comprehension. For this purpose, we present the following classification program for acetabular fractures: Step 1: CT scanning and 3D reconstruction; Step 2: evaluation of acetabular three-column classification; Step 3: assessment of fracture with or without QLP involvement; Step 4: evaluation of QLP four-point classification (Fig. 6).

The successful prognosis of acetabular fractures is highly dependent on the quality of fracture reduction, which in turn, is closely linked to the surgical approach taken. Surgeons need to choose a surgical approach that provides adequate exposure to the fracture site for efficient reduction and fixation, allowing for the restoration of normal femoral head and acetabulum relationships. Additionally, the chosen surgical approach must minimize the risk of iatrogenic injury to surrounding neurovascular structures. It should also reduce any unnecessary stripping of pelvic ligaments and muscle tissue to lower the incidence of surgical complications [19]. In the 3-column classification, the surgical approach for acetabular fractures differs depending on the specific column involved. Anterior column or anterior wall fractures can be addressed through the ilioinguinal or the Stoppa approach [11]. Posterior column or posterior wall fractures can be managed using the Kocher-Langenbeck (KL) approach [11]. Roof column or roof wall fractures can be treated using the iliac fossa, iliofemoral, KL or combined approaches [11].

For patients combined with a QLP fracture, the approach is slightly different according to the fracture pattern. The ilioinguinal approach provides sufficient exposure to the iliac fossa, anterior column, and anterior wall [20]. In cases of simple or incomplete fractures of the QLP, indirect reduction can be achieved by reducing the column. Nonetheless, treating completely fractured or comminuted QLP fractures poses significant challenges. Compared to the traditional ilioinguinal approach, the modified Stoppa approach offers several advantages. It avoids the need for dissection of the inguinal canal, iliac vessels, and sciatic nerve, allowing for improved visibility of the QLP, medial wall of the acetabulum, and sacroiliac joint [21]. However, in obese patients, the modified Stoppa approach faces a challenge due to the obstruction posed by the rectus abdominis muscle, which limits the surgical space available for handling the QLP. Even so, A recent meta-analysis revealed that the modified Stoppa approach results in shorter operation times, reduced intraoperative blood loss, fewer overall complications, and a lower postoperative infection rate [22]. Moreover, the modified Stoppa approach stands out due to its effectiveness in enhancing the visualization of lateral compression injuries and enabling the treatment of both-column fractures through a single incision [23]. Given the convenience of the modified Stoppa approach, it can be considered a viable alternative to the ilioinguinal approach [23]. Furthermore, the Anterior Intrapelvic Approach (AIP) provides a direct pathway to the QLP and the medial aspect of the posterior column, similar to the modified Stoppa approach. AIP offers superior coverage and is well-suited for various types of anterior acetabular fractures compared to the modified Stoppa approach. Thus, AIP also serves as a viable and effective alternative to the conventional Ilioinguinal approach for these fractures [24, 25]. The selection of a surgical technique for fractures in the QLP is contingent upon the specific classification of the fracture. It has been observed that type B and C acetabular fractures frequently coincide with QLP fractures. In accordance with the three-column classification system, one or two surgical approaches are typically employed for type B and C fractures to achieve adequate visual access [11]. When addressing fractures that involve the QLP, it is advisable to prioritize the modified Stoppa approach or the AIP approach, based on their individual advantages.

This study has some limitations. Firstly, the sample data for this study is only from a single center, and the 3-column classification of acetabular fractures is too diverse, which may not represent some fracture lines and heat map models. Secondly, considering the individual differences in skeletal anatomical morphology, although we have expanded the radiation range for converting fracture lines into heat maps, there may still be deviations from the actual results.

## Conclusions

The adoption of a three-column classification system for assessing acetabular fractures is considered reliable and comprehensive. Among this classification, the QLP fractures are commonly observed in type B and C. It is important to carefully identify these fractures during the diagnostic process. Therefore, based on the three-column classification, we have amalgamated QLP fractures and formulated a classification program for acetabular fractures. This integration enables us to provide potential guidance for the overall classification and surgical treatment strategies for acetabular fractures.

## Data Availability

The datasets used and/or analysed during the current study are available from the corresponding author on reasonable request.

## References

[1] Ferguson TA, Patel R, Bhandari M, Matta JM (2010). Fractures of the acetabulum in patients aged 60 years and older: an epidemiological and radiological study. J Bone Joint Surg Br.

[2] Butterwick D, Papp S, Gofton W, Liew A, Beaulé PE (2015). Acetabular fractures in the elderly: evaluation and management. J Bone Joint Surg Am.

[3] Tile Marvin HDL, Kellam James F, Vrahas M (2015). Fractures of the pelvis and acetabulum: principles and methods of management.

[4] ElNahal WA, Abdel Karim M, Khaled SA, Abdelazeem AH, Abdelazeem H (2018). Quadrilateral plate fractures of the acetabulum: proposition for a novel classification system. Injury.

[5] Polesello GC, Nunes MA, Azuaga TL, de Queiroz MC, Honda EK, Ono NK (2012). Comprehension and reproducibility of the Judet and Letournel classification. Acta Ortop Bras.

[6] Herman A, Tenenbaum S, Ougortsin V, Shazar N (2018). There is no column: a new classification for Acetabular fractures. J Bone Joint Surg Am.

[7] Letournel E (1980). Acetabulum fractures: classification and management. Clin Orthop Relat Res.

[8] Judet R, Judet J, Letournel E. Fractures of the Acetabulum (1964). classification and surgical approaches for open reduction. preliminary report. J Bone Joint Surg Am.

[9] Beaulé PE, Dorey FJ, Matta JM (2003). Letournel classification for acetabular fractures. Assessment of interobserver and intraobserver reliability. J Bone Joint Surg Am.

[10] Hurson C, Tansey A, O’Donnchadha B, Nicholson P, Rice J, McElwain J (2007). Rapid prototyping in the assessment, classification and preoperative planning of acetabular fractures. Injury.

[11] Zhang R, Yin Y, Li A, Wang Z, Hou Z, Zhuang Y (2019). Three-column classification for Acetabular fractures: introduction and reproducibility Assessment. J Bone Joint Surg Am.

[12] Ye P, Guo J, Tian S, Wang Z, Li J, Zhao R (2022). Is the T-shaped acetabular fracture really likes a T? A study based on three-dimensional fracture mapping. Injury.

[13] Yang Y, Zou C, Fang Y (2019). A study on fracture lines of the quadrilateral plate based on fracture mapping. J Orthop Surg Res.

[14] Cimerman M, Kristan A, Jug M, Tomaževič M (2021). Fractures of the acetabulum: from yesterday to tomorrow. Int Orthop.

[15] Schwabe P, Wichlas F, Druschel C, Jacobs C, Haas NP, Schaser KD (2014). Komplikationen Nach Osteosynthetischer Versorgung Von azetabulumfrakturen [Complications after osteosynthetic treatment of acetabular fractures]. Orthopade.

[16] Chen K, Yang F, Yao S, Xiong Z, Sun T, Guo X (2020). Biomechanical comparison of different fixation techniques for typical Acetabular fractures in the Elderly: the role of Special Quadrilateral Surface buttress plates. J Bone Joint Surg Am.

[17] Prasartritha T, Chaivanichsiri P (2013). The study of broken quadrilateral surface in fractures of the acetabulum. Int Orthop.

[18] Chen K, Yao S, Yin Y, Wan Y, Ahn J, Zhu S (2023). A new classification for quadrilateral plate fracture of acetabulum. Injury.

[19] Mayo KA (1990). Surgical approaches to the acetabulum. Techniques Orthop.

[20] Letournel E (2019). Acetabulum fractures: classification and management. J Orthop Trauma.

[21] Ponsen KJ, Joosse P, Schigt A, Goslings JC, Luitse JS (2006). Internal fracture fixation using the Stoppa approach in pelvic ring and acetabular fractures: technical aspects and operative results. J Trauma.

[22] Srivastava A, Rajnish RK, Kumar P, Haq RU, Dhammi IK (2023). Ilioinguinal versus modified Stoppa approach for open reduction and internal fixation of displaced acetabular fractures: a systematic review and meta-analysis of 717 patients across ten studies. Arch Orthop Trauma Surg.

[23] Al Adawy AS, Aziz AHA, El Sherief FA, Mahmoud WS, Mabrook M, Hassan YE (2020). Modified Stoppa as an alternative surgical approach for fixation of anterior fracture acetabulum: a randomized control clinical trial. J Orthop Surg Res.

[24] Ciolli G, De Mauro D, Rovere G, Smakaj A, Marino S, Are L, et al. Anterior intrapelvic approach and suprapectineal quadrilateral surface plate for acetabular fractures with anterior involvement: a retrospective study of 34 patients. BMC Musculoskelet Disord. 2021;22(S2). 10.1186/s12891-021-04908-z PMC8717694 34969392

[25] Trikha V, Das S, Aruljothi V, Chowdhury B (2020). Prospective evaluation of outcome of Acetabular fractures managed by Anterior Intrapelvic Approach. Indian J Orthop.

